# Dysfunction in atox-1 and ceruloplasmin alters labile Cu levels and consequently Cu homeostasis in *C. elegans*

**DOI:** 10.3389/fmolb.2024.1354627

**Published:** 2024-02-08

**Authors:** Ann-Kathrin Weishaupt, Karsten Lamann, Elke Tallarek, Aidan T. Pezacki, Carson D. Matier, Tanja Schwerdtle, Michael Aschner, Christopher J. Chang, Stephen R. Stürzenbaum, Julia Bornhorst

**Affiliations:** ^1^ Food Chemistry, Faculty of Mathematics and Natural Sciences, University of Wuppertal, Wuppertal, Germany; ^2^ TraceAge – DFG Research Unit on Interactions of Essential Trace Elements in Healthy and Diseased Elderly (FOR 2558), Berlin-Potsdam-Jena-Wuppertal, Germany; ^3^ Tascon GmbH, Münster, Germany; ^4^ Departments of Chemistry and Molecular and Cell Biology, University of California, Berkeley, Berkeley, CA, United States; ^5^ German Federal Institute for Risk Assessment (BfR), Berlin, Germany; ^6^ Department of Molecular Pharmacology, Albert Einstein College of Medicine, Bronx, NY, United States; ^7^ Department of Analytical, Environmental and Forensic Sciences, School of Cancer & Pharmaceutical Sciences, Faculty of Life Sciences and Medicine, King’s College London, London, United Kingdom

**Keywords:** copper, homeostasis, total vs. labile copper, ToF-SIMS, *C. elegans*

## Abstract

Copper (Cu) is an essential trace element, however an excess is toxic due to its redox properties. Cu homeostasis therefore needs to be tightly regulated via cellular transporters, storage proteins and exporters. An imbalance in Cu homeostasis has been associated with neurodegenerative disorders such as Wilson’s disease, but also Alzheimer’s or Parkinson’s disease. In our current study, we explored the utility of using *Caenorhabditis elegans* (*C. elegans*) as a model of Cu dyshomeostasis. The application of excess Cu dosing and the use of mutants lacking the intracellular Cu chaperone atox-1 and major Cu storage protein ceruloplasmin facilitated the assessment of Cu status, functional markers including total Cu levels, labile Cu levels, Cu distribution and the gene expression of homeostasis-related genes. Our data revealed a decrease in total Cu uptake but an increase in labile Cu levels due to genetic dysfunction, as well as altered gene expression levels of Cu homeostasis-associated genes. In addition, the data uncovered the role ceruloplasmin and atox-1 play in the worm’s Cu homeostasis. This study provides insights into suitable functional Cu markers and Cu homeostasis in *C. elegans*, with a focus on labile Cu levels, a promising marker of Cu dysregulation during disease progression.

## 1 Introduction

**FIGURE 1:**
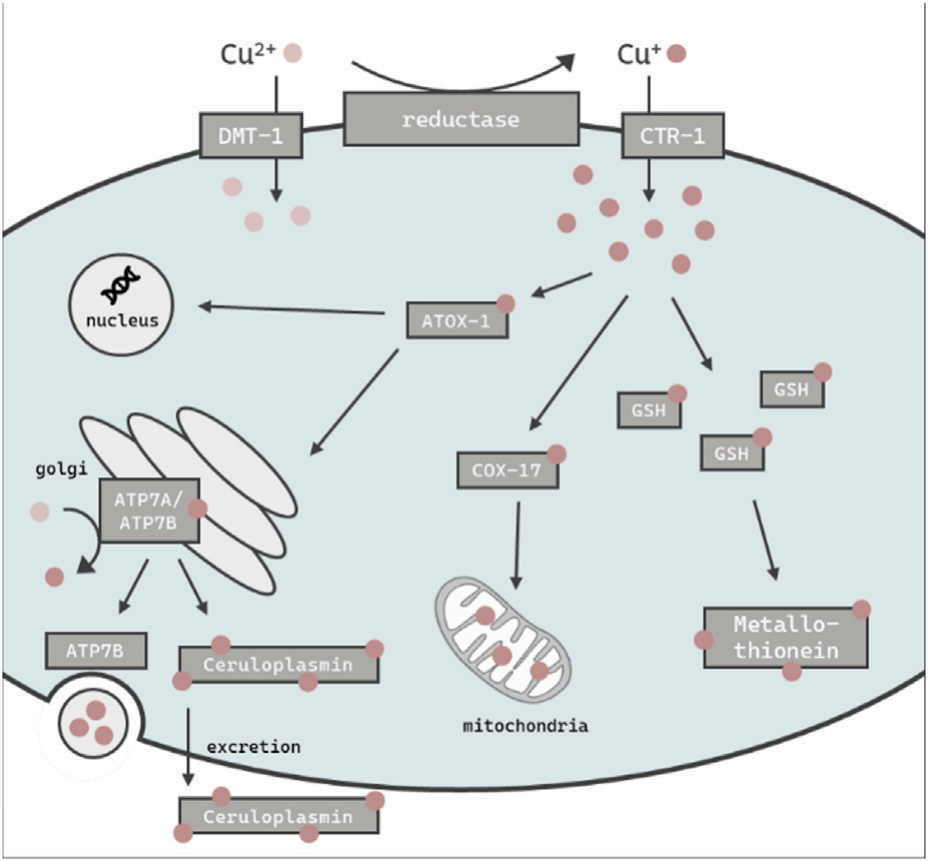
Schematic overview of the assumed intracellular Cu import, distribution, storage and excretion in *C. elegans*. Cu is taken up primarily as Cu^+^ via CTR-1 or alternately as Cu^2+^ via DMT-1. ATOX-1 mediates Cu distribution to the golgi, nucleus or mitochondria via COX-17. GSH and metallothionein is thought to be involved in the chelating of excess Cu, while the majority is stored in the ceruloplasmin. The efflux of excess Cu is mediated by ATP7B via vesicular sequestration.

The essential trace element and micronutrient copper (Cu) functions as a catalytic cofactor for a variety of enzymes in biological processes, including mitochondrial respiration and the synthesis of biocompounds (Chen et al., 2022). Cu is widely used in industry and agriculture, both a major contributor of soil and water pollution (Li et al., 2021; Vázquez-Blanco et al., 2020). Excess in Cu (beyond the physiological need) can be harmful to organisms due to its redox properties and the ability to promote the formation of reactive oxygen species (ROS) (An et al., 2022). In humans, altered Cu levels lead to oxidative stress and in consequence can result in the onset of neurodegenerative disorders (Que et al., 2008), as well as cancer (Ge et al., 2022). Therefore, the tightly controlled homeostasis of Cu levels is of importance to cellular and organismal wellbeing (Bisaglia and Bubacco, 2020). Mammals and other organisms are therefore endowed with a complex network of proteins which are involved in the regulation of Cu homeostasis. These proteins work in concert to coordinate the import, export and intracellular utilization of Cu, thus maintaining cellular levels within a specific range, thereby preventing the consequences of Cu overload (Chen et al., 2022). The reduced form of Cu (Cu^+^) enters the cell mainly in via a high affinity copper uptake protein (CTR-1) dependent on intracellular Cu levels (Clifford et al., 2016), while oxidized Cu^2+^ is taken up via the divalent metal transporter 1 (DMT-1) (Shawki et al., 2015) (Figure 1). Antioxidant protein 1 (ATOX-1), a Cu metallochaperone protein which obtains Cu from CTR-1, engages in the intracellular transport of Cu to target organelles such as the nucleus or golgi (Kamiya et al., 2018). As Cu serves as a cofactor for a variety of mitochondrial enzymes, the cytochrome c oxidase copper chaperone (COX-17) regulates mitochondrial Cu import (Punter et al., 2000). The major Cu-carrier in the blood is the multifunctional protein ceruloplasmin, which stores up to 90% of total Cu (Hellman and Gitlin, 2002) and displays ferroxidase activity (Linder, 2016). Furthermore, ceruloplasmin serves as an extracellular scavenger for reactive species and therefore limits oxidative damage (Linder, 2016). Likewise, metallothioneins bind metal ions like cadmium, zinc and Cu for detoxification and protection against oxidative stress (Höckner et al., 2011). Cu excretion is mediated by ATP7B, which either delivers Cu to ceruloplasmin (Weiss et al., 2008) for subsequent elimination via the bile (Prohaska, 2008) or translocates from the golgi to the plasma membrane to efflux Cu via vesicular sequestration (Monty et al., 2005; Cater et al., 2006). To date, serum or plasma Cu status is derived solely by determining total Cu or ceruloplasmin levels (Olivares et al., 2008; Hackler et al., 2020). Cellular copper is partitioned between tightly-bound pools in cuproenzymes, which bind copper with K_d_ values in the 10^−15^ M and tighter, and labile pools, defined as loosely bound to low-molecular weight ligands with K_d_ values that are orders of magnitude weaker, typically in the 10^−10^ to 10^−14^ M range, which can regulate diverse transition metal signaling processes (Banci et al., 2010; Carter et al., 2014; Cotruvo et al., 2015; Hare et al., 2015; Ackerman et al., 2017; Ackerman and Chang, 2018). The labile Cu fraction provides an estimation of Cu activity and may thus serve as a better functional marker than total Cu as Cu participates in transition metal signaling pathways beyond traditional roles in metabolism (Chang, 2015; Pham and Chang, 2023). Indeed, labile Cu was recently identified as a marker for the Cu status in human serum (Schwarz et al., 2023; Tuchtenhagen et al., 2023). This study aimed to further our knowledge base regarding Cu homeostasis and dyshomeostasis, with a particular focus on labile Cu levels. This will shed light on the regulatory mechanisms involved when an organism is challenged with an oversupply of total Cu and/or labile Cu, respectively. For this purpose, we use the model organism *Caenorhabditis elegans* (*C. elegans*), which is an *in vivo* invertebrate model organism suitable to study metal homeostasis and toxicity (Aschner et al., 2017). An additional advantage of using *C. elegans* is the wide range of available deletion (Δ) mutants. Although the metallomic underpinning of Cu homeostasis in *C. elegans* shares many homologies to mammals, studies using the nematode in research on Cu homeostasis are scarce (Wakabayashi et al., 1998; Chun et al., 2017; Yuan et al., 2018). Here, we studied Cu dyshomeostasis by excess Cu feeding as well as by using models displaying genetic Cu disbalance, such as the mutant ceruloplasminΔ, which lacks the major Cu storage protein, as well as an atox-1Δ mutant. Taken together, we define the role of ceruloplasmin and atox-1 in Cu homeostasis and identify suitable functional markers in the model *C. elegans*.

## 2 Materials and methods

### 2.1 *C. elegans* handling and Cu treatment

*C. elegans* strain Bristol N2 (wildtype) and deletion mutants *mtl-2*(gk125) were obtained from the *Caenorhabditis* Genetics Center (CGC, Minneapolis, USA), which is funded by the National Institutes of Health Office of Research Infrastructure Programs. Deletion mutants *atox-1* (tm1220), *mtl-1*(tm1770) and the ceruloplasmin mutant (tm14205) were obtained from the Mitani laboratory at Tokyo Women’s Medical University. Worm strains *mtl-1;mtl-2*(zs1), and P*mtl-1*::GFP and P*mtl-2*::mcherry (integrated into the genome by Mos1-mediated single-copy insertion (MosSCI)) were generated by the Stephen Stürzenbaum laboratory. Note, the P*mtl-2*::mcherry strain contained an addition nuclear localization signal (NLS). All strains were cultivated on agar plates coated with *Escherichia coli* (*E. coli*) at 20 °C as previously described (Brenner, 1974). Worms were synchronized as described in (Porta-de-la-Riva et al., 2012) and placed on NGM plates until L4 larval stage. L4 stage worms were treated with copper-enriched inactivated *E. coli* (OP50) on NGM plates for 24 h. The bacteria were inactivated for 4 h at 70°C (Baesler et al., 2021). Stock solutions of CuSO_4_ (≥99.99%, Sigma Aldrich) were prepared fresh in bidistilled water.

### 2.2 Lethality studies after Cu exposure

For lethality testing, worms were counted manually as alive or dead after 24 h of Cu exposure. Worms were defined as dead if they demonstrated no movement after prodding with a platinum wire.

### 2.3 Total Cu quantification via ICP-OES

Total Cu content in worms was quantified using inductively coupled plasma-optical emission spectrometry (ICP-OES) (Avio 220 Max, Perkin Elmer). Following 24 h Cu exposure, 1000 worms per condition were washed 4x using 85 mM NaCl + 0.01% Tween 20, pelleted by centrifugation, frozen in liquid nitrogen and stored at −80°C. Pellets were homogenized using an ultrasonic probe (UP100H, Hielscher) and subsequently dried at 95°C. Yttrium (Y) (ROTI^®^STAR, Carl Roth) was added as internal standard and the samples were digested at 95°C using 500 µL of a 1:1 mixture (v:v) of HNO_3_ (Suprapur^®^, Merck KGaA) and H_2_O_2_ (for ultratrace analysis, Sigma Aldrich) and re-dissolved in 2% HNO_3_. The following parameters were used for measurements: Plasma power: 1500 W, cooling gas: 8 L/min, auxiliary gas: 0.2 L/min, nebulizer (MicroMist™) gas: 0.7 L/min, wavelengths: Cu – 327.939 and Y – 371.029. The analysis was performed using external calibration (multi element mix (spectec-645) + Y) and verified by measuring certified reference material BCR^®^-274 (Single cell protein, Institute for Reference Materials and Measurement of the European Commission) and SRM^®^-1643f (Trace Elements in Natural Water, National Institute of Standards and Technology). The Cu content was normalized to the protein amount determined using a BCA assay (Walker, 1994) using bovine serum albumin (Sigma Aldrich) for external calibration.

### 2.4 Quantification of labile Cu by fluorescent dye CF4

Labile Cu levels were assessed using the fluorescent dye Copper Fluor-4 (CF4), which has an apparent K_d_ value of 2.9 × 10^−13^ M for a 1:1 copper:probe stoichiometry that is well-matched to monitor labile Cu pools by reversible Cu binding without depleting the total Cu stores (Xiao et al., 2018). Stock solutions were prepared in DMSO (5 mM). Following Cu treatment, worms were exposed to 10 µM CF4 for 3 h in the dark in incubation buffer (25 mM HEPES, 120 mM NaCl, 5.4 mM KCl, 5 mM Glucose, 1.3 mM CaCl_2_, 1 mM MgCl_2_, 1 mM NaH_2_PO_4_, pH = 7.35, 0.01% Tween 20). Thereafter, fluorescence intensity was assessed by either fluorescence microscopy or plate reader measurement. Worms were transferred to 4% agarose pads on microscope slides and anesthetized by 5 mM levamisole (Sigma Aldrich). Fluorescence images as well as intensities were obtained with a DM6 B fluorescence microscope and the Leica LAS X software (Leica Microsystems GmbH) using a triple band excitation filter and constant settings as well as light exposure times. 400 worms per well in triplicates were transferred into a 96 well plate for plate reader measurements, while another aliquot was stored for protein measurement. Bottom reads were performed using a microplate reader Infinite^®^ M Plex (Tecan) with wavelengths of 415 nm for excitation and 660 nm for emission.

### 2.5 Cu imaging by ToF-SIMS

Worms were incubated with 2 mM CuSO_4_, following 3x washing steps with 85 mM NaCl and 3x washing steps with Rotipuran Ultra (Carl Roth). Subsequently, about 20 worms per strain were transferred to indium tin oxide (ITO) coated glass slides. In order to locate the 3-dimensional Cu distribution in non-fluorescent-labeled worms, ToF-SIMS 3D depth profiling analysis was performed using an IONTOF “ToF.SIMS^5^”. Sputtering was performed using a O_2_^+^, 2 keV ion beam with a maximum current of 650 nA rastered across 700 × 700 μm^2^. Analysis was performed using a Bi_1_^+^, 30 keV, 0.5 pA ion beam in spectrometry mode, rastered across 500 × 500 μm^2^ within the center of the sputter crater. Secondary ions of positive polarity were mass analyzed.

### 2.6 Gene expression via quantitative real-time PCR analysis

Total RNA content was isolated using the Trizol method, published by Bornhorst et al. (2014), of which 1 µg was transcribed using the High Capacity cDNA Reverse Transcription Kit (Applied Biosystems, Thermo Fisher Scientific) following the manufacturer’s protocol. Quantitative real-time PCR was carried out on the AriaMx Real-Time PCR System in duplicate wells for each gene using TaqMan Gene Expression Assay probes (Applied Biosystems, Thermo Fisher Scientific) according the manufacturer’s instructions. The *AFDN* homolog *afd-1* was used as housekeeping gene for normalization by the comparative 2^−ΔΔCt^ method (Livak and Schmittgen, 2001). The probes used were: *afd-1* (Ce0241573_m1), *ctr-1* (Ce02417730_g1), *mtl-1* (Ce02551471_s1), *mtl-2* (Ce0251627_s1), *cua-1* (Ce02454392_m1), *cuc-1* (Ce02449329_g1), *f21d5.3/ceruloplasmin* (Ce02456979_m1) and *cox-17* (Ce02442285_m1).

### 2.7 Metallothionein expression

To assess metallothionein expression, P*mtl-1*::GFP and P*mtl-2*::mcherry transgenes were used. After Cu treatment and 4x washing steps with 85 mM NaCl + 0.01% Tween 20, excess liquid was aspirated to yield 1600 worms in 400 µL. 3 × 100 µL were transferred as triplicate into a 96 well plate, the remaining 100 µL were used for protein quantification. Bottom read measurements were conducted at 488 nm (excitation) and 509 nm (emission) for GFP-tagged worms and 561 nm (excitation) and 610 nm (emission) for mcherry-tagged worms using a Tecan microplate reader Infinite^®^ M Plex (Tecan, Switzerland). Additionally, worms were transferred to 4% agarose pads on microscope slides, followed by anesthesia using 5 mM levamisole (Sigma Aldrich). Images were taken using a Leica DM6 B fluorescence microscope (Leica Microsystems GmbH) with constant settings and light exposure time.

### 2.8 Statistical analysis

Statistical analyses were carried out with GraphPad Prism 6 (GraphPad Software, La Jolla, CA, USA). Statistical tests and significance levels are listed in figure captions.

## 3 Results

### 3.1 Lethality after Cu exposure

**FIGURE 2:**
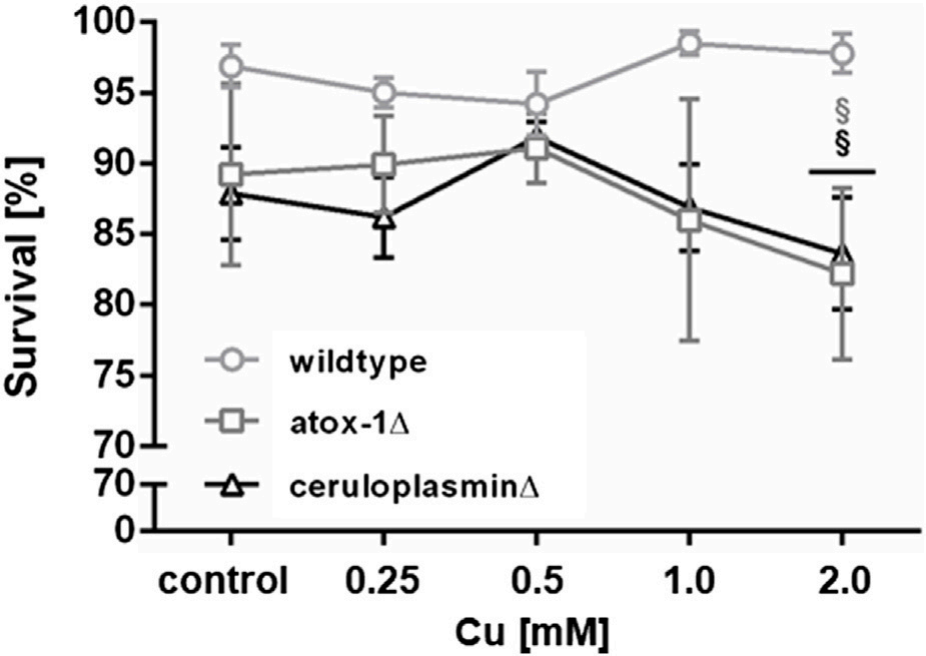
Lethality in wildtype worms, atox-1Δ and ceruloplasminΔ deletion mutants following an exposure to Cu for 24 h at age-synchronized L4 larvae stage. Data presented are mean values of n ≥ 4 experiments ± SEM. Statistical analysis using 2-way ANOVA with Tukey’s multiple comparison. Significance level with *α* = 0.05: §: *p* ≤ 0.05 compared to wildtype in same condition.

Lethality testing following 24 h Cu exposure revealed no toxic effect up to 2 mM in wildtype worms, while atox-1Δ and ceruloplasminΔ deletion mutants presented a significant reduction of survival of about 10% after 2 mM Cu treatment (Figure 2). During lethality testing we noticed that worms exposed to 2 mM Cu started to display shortened and thinner phenotype, which indicated the onset of a developmental delay. Concentrations above 2 mM were not considered, since worms were previously shown to avoid higher amounts of Cu, as described in Guo et al. (2015) and Munro et al. (2020).

### 3.2 Total Cu vs. labile Cu levels

**FIGURE 3:**
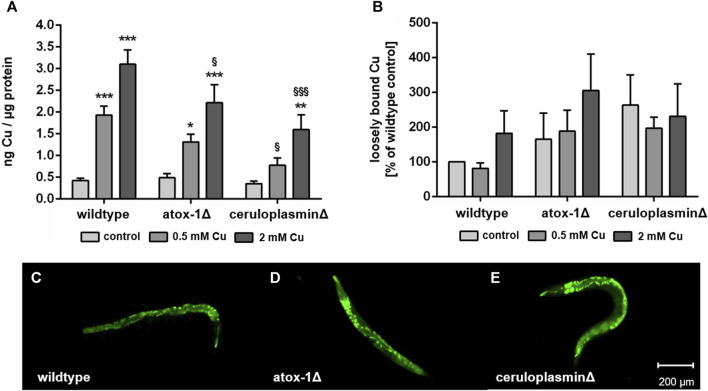
**(A)** Total Cu levels quantified by ICP-OES and **(B)** labile Cu levels assessed by using fluorescent dye Copper Fluor-4 (CF4). Displayed are representative images of **(C)** wildtype worms, **(D)** atox-1- and **(E)** ceruloplasmin-deficient worms after CF4 treatment (control, not treated with Cu). Data presented are mean values of n ≥ 4 independent experiments + SEM. Statistical analysis using 2-way ANOVA with Tukey’s multiple comparison. Significance level with *α* = 0.05: *: *p* ≤ 0.05; **: *p* ≤ 0.01; ***: *p* ≤ 0.001 compared to untreated control and §: *p* ≤ 0.05 and §§§: *p* ≤ 0.001; compared to wildtype under the same condition.

Following 24 h of treatment with CuSO_4_-enriched *E. coli* up to 2 mM, total Cu levels of wildtype, atox-1Δ and ceruloplasminΔ deletion mutants were quantified by ICP-OES (Figure 3A). Cu basal levels were indistinguishable in all 3 worm strains with 0.42 ± 0.05 ng Cu per µg protein in wildtype worms respectively. In addition, a concentration-dependent increase in Cu levels was observed for all strains. However, mutants with impaired Cu homeostasis displayed significantly lower total Cu levels than wildtype worms, in particular ceruloplasmin-deficient worms. Labile Cu levels were determined by fluorescent dye CF4 (Figure 3B). Labile Cu levels tended to be elevated following Cu treatment of wildtype and atox-1Δ worms, furthermore, a higher basal level of labile Cu levels was observed in untreated ceruloplasmin-deficient worms. In general, labile Cu levels appeared to be higher in worms characterized by a disturbed Cu homeostasis (Figures 3C–E).

### 3.3 Cu imaging and depth profiling by ToF-SIMS

**FIGURE 4:**
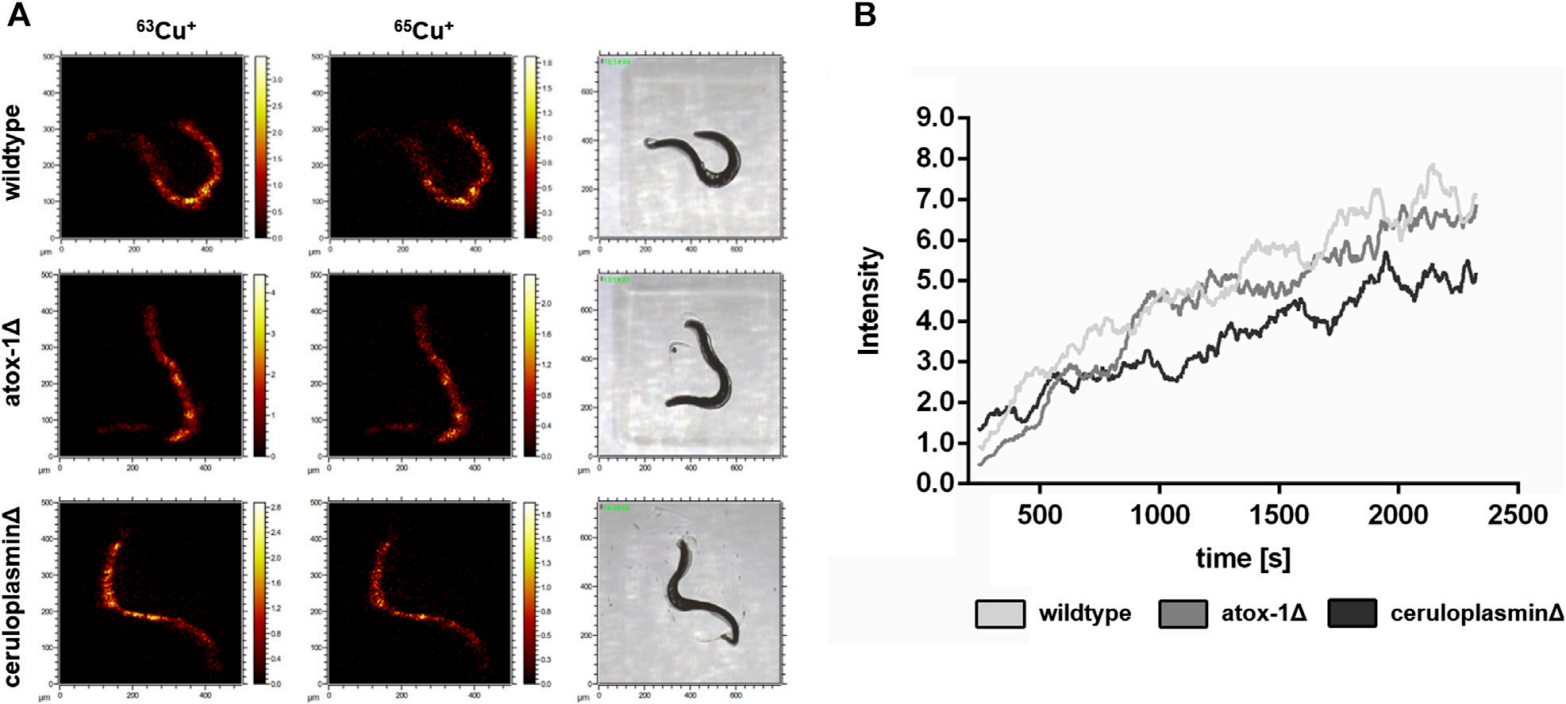
ToF-SIMS analysis of wildtype, atox-1Δ and ceruloplasminΔ worms following 2 mM Cu treatment for 24 h. **(A)** Distribution of ^63^Cu^+^ and ^65^Cu^+^ over the total sputtered depth (excluding the first 250 sputter seconds). **(B)** Depth distribution of ^63^Cu^+^ for all 3 worm strains (O_2_^+^ sputtering) covering approximately half of the worm’s depth.

The location of Cu in worms exposed to 2 mM Cu for 24 h was investigated by Time-of-Flight Secondary Ion Mass Spectrometry (ToF-SIMS). 3D depth profiles were created to determine the Cu distribution in relation to the worms’ depth. In “dual-beam-mode” of ToF-SIMS depth profiling each sample surface was continuously sputtered by an ion beam (O_2_^+^), while a second ion beam (Bi^+^) was used to image the respective intensity of Cu in the resulting crater surface (Figure 4A). Subsequently, the lateral distribution over the total sputtered depth excluding the first sputter seconds in order to exclude surface contaminants as well as the regional depth profile at the worm positions were calculated from the ToF-SIMS raw data stream. Images of the isotopes ^63^Cu^+^ and ^65^Cu^+^ (Figure 4A) were comparable in distribution. Figure 4A shows that the highest Cu intensity is located in the middle part of the worm corpus for all three strains. The highest Cu intensity was detected in wildtype worms, whereas the ceruloplasmin-deficient worms demonstrated the lowest Cu intensity (Figure 4B).

### 3.4 Gene expression of Cu homeostasis-related genes upon Cu exposure

**FIGURE 5:**
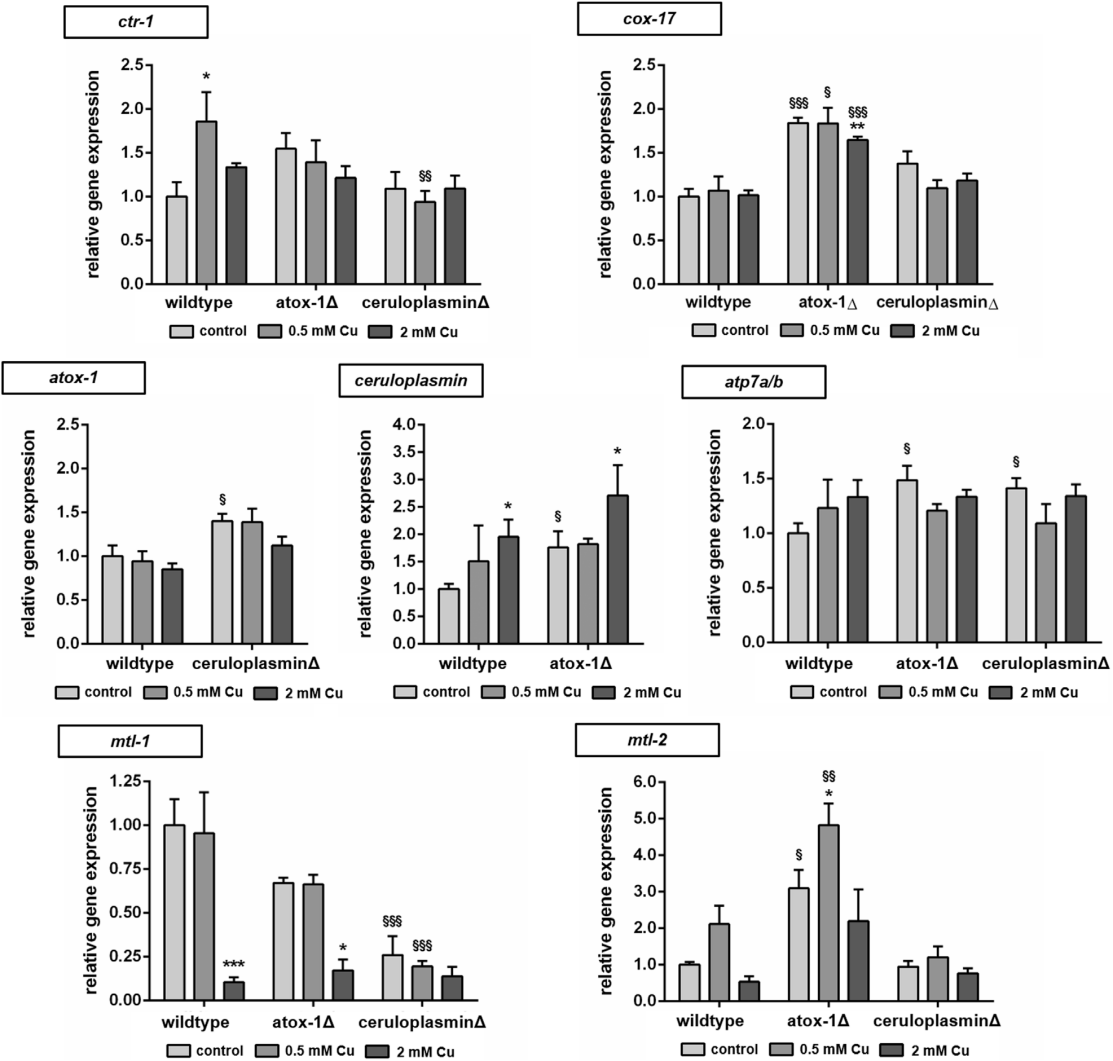
Relative mRNA levels of Cu transport and storage-related genes following 24 h Cu treatment determined by RT-qPCR normalized to *afd-1* (*AFDN* homologue) as housekeeper. Data presented are mean values of n = 4 independent experiments + SEM. Statistical analysis using 2-way ANOVA with Tukey’s multiple comparison. Significance level with *α* = 0.05: *: *p* ≤ 0.05; **: *p* ≤ 0.01; ***: *p* ≤ 0.001 compared to untreated control and §: *p* ≤ 0.05; §§: *p* ≤ 0.01 and §§§: *p* ≤ 0.001 compared to wildtype under the same condition.

The relative mRNA levels of Cu transport- and storage-related genes were determined via RT-qPCR in wildtype and mutants treated with Cu for 24 h (Figure 5). Target genes were Cu importer *ctr-1* (ortholog to human high affinity copper uptake protein 1 encoded by *SLC31A1*), cytochrome c oxidase copper chaperone *cox-17*, intracellular transporter *atox-1*, *atp7a/b* (ortholog of human *ATP7A* and *ATP7B*) and storage-related genes *ceruloplasmin*, *mtl-1* and *mtl-2* (orthologs to human metallothionein *MT1A* and *MT2A*). In wildtype worms, Cu treatment resulted in an upregulation of *ctr-1*, while atox-1Δ worms displayed already elevated basal levels. Mitochondrial Cu importer *cox-17* expression was elevated in atox-1Δ deletion mutants at the basal level as also following Cu exposure. *Atox-1* mRNA levels did not increase due to Cu exposure but were altered in ceruloplasminΔ worms. Mammalian genomes encode for two isoforms per Cu exporter (*ATP7A* and *ATP7B*), whilst *C. elegans* carries only a single gene of *atp7a/b*, albeit with high sequence similarity to human homologs (Chun et al., 2017). Cu treatment lead to an increase in *atp7a/b* mRNA levels in wildtype worms, which were already significantly elevated in both untreated deletion mutants. Gene expression of *ceruloplasmin* was amplified due to Cu treatment, in addition, atox-1Δ worms displayed significantly higher levels in untreated controls compared to wildtype worms. mRNA levels of *mtl-1* were significantly reduced by about 90% in wildtype worms upon treatment with 2 mM Cu. The basal level of *mtl-1* was lower in atox-1Δ and ceruloplasminΔ deletion mutants (compared to wildtype) but exposure to 2 mM Cu lowered *mtl-1* gene expression further. The expression of *mtl-2* increased at low level exposures (0.5 mM Cu) but reduced at the higher exposure concentration (2 mM), this trend was observed in wildtype and the two deletion mutants, but the expression levels were notably higher in the atox-1Δ mutant.

### 3.5 Metallothionein expression and alterations of Cu uptake in mtl-KO mutants

**FIGURE 6:**
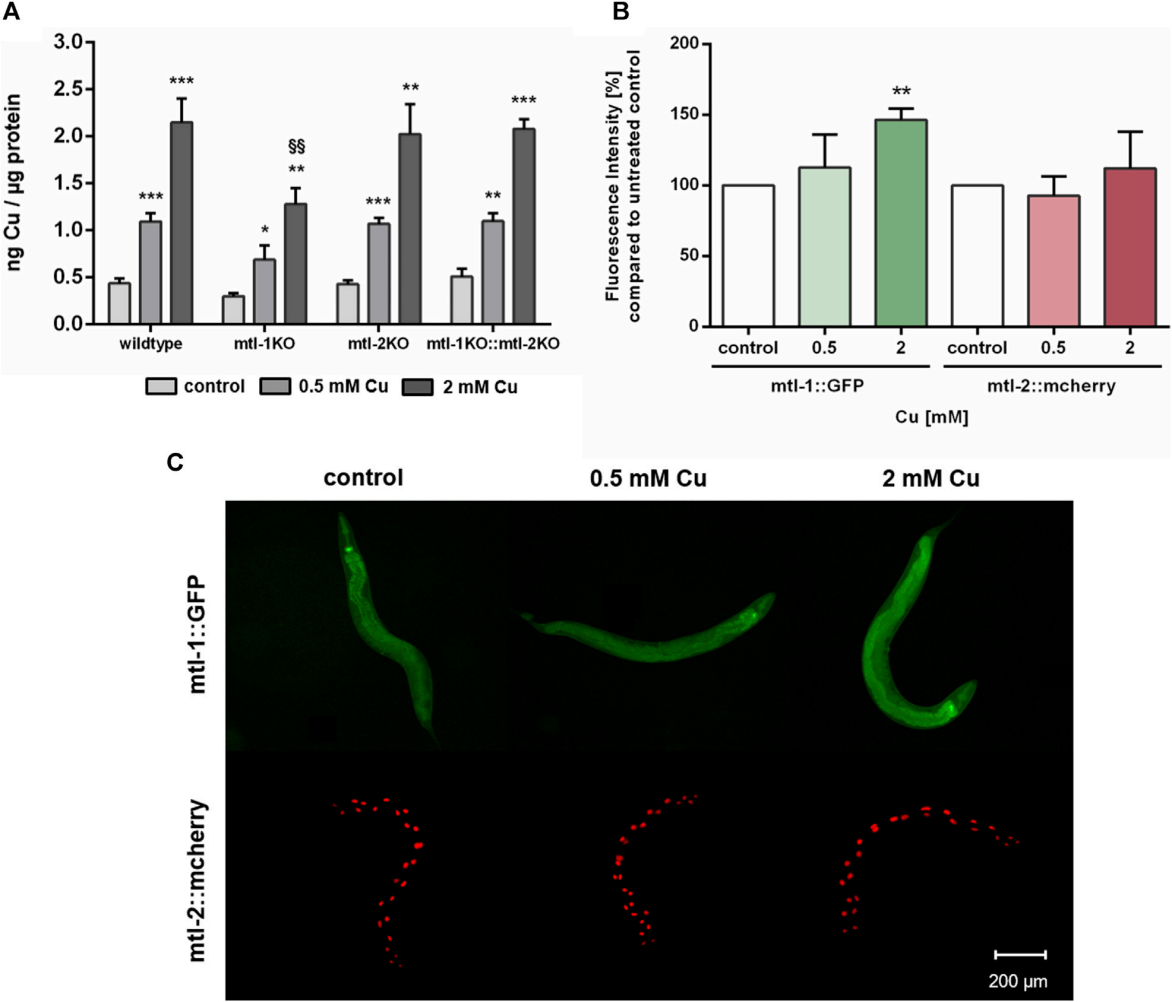
**(A)** Total Cu levels quantified by ICP-OES in wildtype, and the metallothionein knockout strains (*mtl-1*(tm1770), *mtl-2*(gk125) and the double knockout *mtl-1;mtl-2*(zs1)). **(B)** Fluorescence intensity [%] compared to untreated control in P*mtl-1*::GFP and P*mtl-2*::mcherry worms as well as **(C)** representative fluorescence microscopy images. Data presented are mean values of *n* = 4 independent experiments + SEM. Statistical analysis using 2-way ANOVA with Tukey’s multiple comparison. Significance level with *α* = 0.05: *: *p* ≤ 0.05; **: *p* ≤ 0.01; ***: *p* ≤ 0.001 compared to untreated control and §§: *p* ≤ 0.01 compared to wildtype under the same condition.

Since Cu oversupply resulted in decrease of *mtl-1* and *mtl-2* expression, the involvement of metallothionein in Cu homeostasis was further investigated. Therefore, single knockout mutants of mtl-1 (*mtl-1*(tm1770)) and mtl-2 (*mtl-2*(gk125)), as well as the double knockout mutant (*mtl-1*;*mtl-2*(zs1) were incubated with Cu as described and total Cu levels were determined by ICP-OES. Results revealed a concentration-dependent Cu uptake for all tested strains, however, mtl-1KO (*mtl-1*(tm1770)) worms displayed significant less Cu uptake after 2 mM CuSO_4_ treatment (Figure 6A), but also lower levels in other trace elements (Supplementary). Although mRNA is required for protein synthesis, it does not inversely dictate that mRNA levels and mRNA induction levels are universally proportional to each other (Buccitelli and Selbach, 2020). Consequently, we investigated the induction of *mtl-1* and *mtl-2* using the fluorescence-tagged transgenes P*mtl-1*::GFP and P*mtl-2*::mcherry, generated by the Mos1-mediated single-copy insertion (MosSCI) techniques, note the latter modified to contain a nuclear localization signal (NLS). Fluorescence plate reader measurements revealed a marginal increase in *mtl-1* expression but *mtl-2* levels remained, at large, unaffected by Cu exposure (Figure 6B), which was also visualized by fluorescence microscopy (Figure 6C).

## 4 Discussion

Cu is an essential trace element, serving as an enzyme cofactor due to its redox properties (Chen et al., 2022). In excess, however, Cu can promotes adverse health effects, which are mainly caused by the excessive formation of reactive oxygen species at the cellular level (Song et al., 2014). Excess Cu, beyond the homeostatic range, has been linked to the onset of numerous neurodegenerative diseases, and foremost Wilsons disease (WD) (Shribman et al., 2021; Squitti et al., 2023). It is therefore of importance to have mechanisms in place that allow an efficient regulation of Cu homeostasis. It is crucial to better understand how Cu homeostasis is balanced, and characterize these regulatory mechanisms. Two key players are ceruloplasmin and atox-1 and the consequences of their loss of function should be investigated. In addition, suitable markers and new tools to assess Cu status are needed, and the nematode *C. elegans* is a powerful model to address these shortcomings.

**FIGURE 7:**
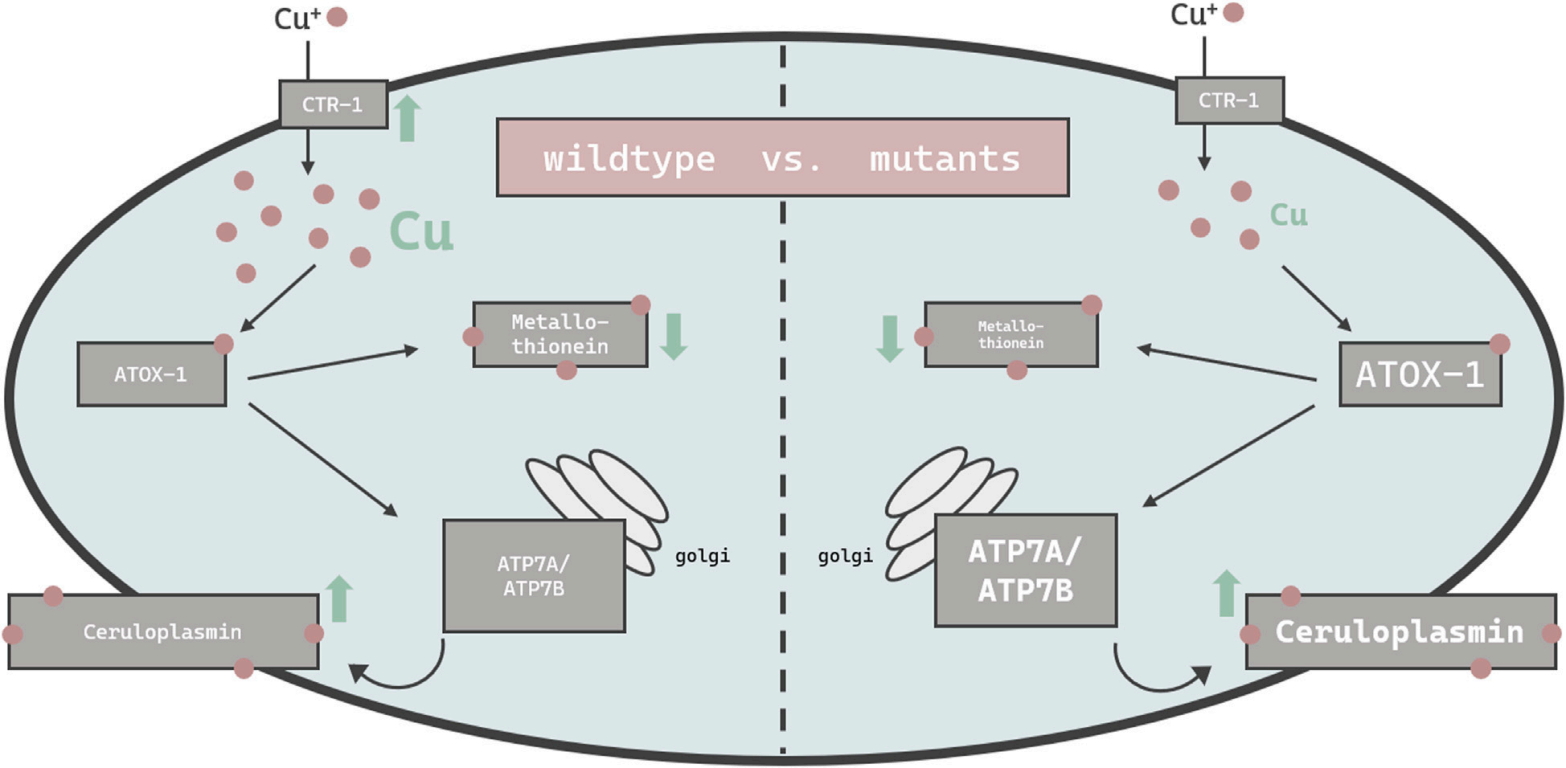
Schematic overview of the changes in the bioavailability and expression of genes responsible for Cu homeostasis in *C. elegans*. Displayed are changes in wildtype worms (left) vs. mutant worms (atox-1Δ or ceruloplasminΔ) (right). Up- and downregulation of mRNA levels by excessive Cu feeding are indicated by green arrows, while differences in basal levels due to genetics compared to wildtype worms is indicated by smaller or larger font size.

Others have demonstrated that high doses of Cu can result in cellular toxicity in different modes of applications (Chun et al., 2017; Yuan et al., 2018; Zhang et al., 2021). Our study focused on metal homeostasis and investigated physiological endpoints rather than toxicology. Accordingly, Cu was applied via *E. coli* on agar plates up to 2 mM for a 24 h duration, which did not impact majorly on lethality rates. Having said that, mutants with disturbed Cu homeostasis presented a reduced survival rate of about 10% and are consequently Cu-hypersensitive. In addition, concentrations above 2 mM were avoided, as worms move away from the exposed *E. coli* and starve (Guo et al., 2015; Munro et al., 2020). Cu^2+^, as used in our study, is reduced to Cu^+^ by a yet unknown reductase in *C. elegans* and subsequently taken up by importer CTR-1. The transcription of *ctr-1* increased in wildtype worms exposed to 0.5 mM Cu, which is in contrast to observations made by Clifford et al. (Clifford et al., 2016), however *ctr-1* expression was not modulated in worms challenged with the higher dosage of Cu (2 mM). Total Cu uptake increased in a concentration-dependent manner (Chun et al., 2017; Yuan et al., 2018), yet significantly less in the atox-1 and ceruloplasmin deletion mutants, suggesting that these mutants are characterized by an altered storage capacity. Factors that may further contribute to a disturbed homeostasis may include a reduced influx, an increased efflux or a lack of sufficient storage capacity, or a combination thereof. Li et al. display normal Cu levels in ceruloplasmin-KO mice in the cerebral cortex and hippocampus (Li et al., 2022). The brain is, after the liver, the organ with the highest Cu occurrence (An et al., 2022). Consequently, we investigated whether Cu accumulates in specific areas of the worm. ToF-SIMS analysis revealed a universal distribution of Cu across the worm body, but it should be noted that neurons are present not only in the head region but over the entire body of the worm (Gendrel et al., 2016). Even if ToF-SIMS analysis goes further than microscopy, as an additional depth profile analysis is included, the resolution is not sufficient to localize Cu within a cell (subcellular). Therefore, future studies should focus on neuronal cells by using techniques such as NanoSIMS (Nano Secondary Ion Mass Spectrometry) (Witt et al., 2020). With respect to the total Cu amount, the ToF-SIMS results matched our ICP-OES data, where the highest Cu concentrations were measured in wildtype worms and the lowest in ceruloplasmin-deficient worms, following a 24 h treatment with 2 mM Cu (Figure 7). Studies in *ATOX-1*KO mice and cell culture revealed a disturbed Cu homeostasis (Hamza et al., 2001; Hamza et al., 2003). Furthermore, Zhang et al. displayed the phenotype of a *C. elegans atox-1*KO model in form of reduced brood size and distal tip cell migration defects (Zhang et al., 2020). However, data on the Cu status were lacking, which are critical for the evaluation of Cu toxicity. In humans, the highest mRNA levels of ATOX-1 in the brain were detected in the cerebral cortex and hippocampus, with elevated ATOX-1 activity due to increased Cu levels (Lutsenko et al., 2010). Moreover, ATOX-1 is thought to possess antioxidative properties (Lutsenko et al., 2010), as increased endogenous ATOX-1 levels protect against oxidative stress and promote neuronal survival (Kelner et al., 2000). In our study, *cox-17* expression was elevated in the atox-1Δ deletion mutant*,* which might indicate an increased Cu transport into mitochondria. Whilst atox-1 participates in intracellular Cu distribution, ceruloplasmin is the major Cu storage protein responsible for the binding of 90% of total Cu (Hellman and Gitlin, 2002). Genetic loss of ceruloplasmin can lead to the autosomal recessive disorder “aceruloplasminemia”, which is characterized by progressive neurodegeneration (Kono, 2012). Elevated Cu or labile Cu levels are not the only concern in aceruloplasminemia observed in aging worms (Muchenditsi et al., 2021). Due to ceruloplasmin’s ferroxidase activity, it is essential for iron (Fe) oxidation during cellular export, resulting in cellular Fe accumulation in aceruloplasminemia (Miyajima, 2015). Despite its importance in Cu storage protein in mammals, to date no research has focused on the role of ceruloplasmin in Cu homeostasis in *C. elegans*. Our data revealed that Cu levels were altered due to Cu supplementation in ceruloplasmin-deficient worms, but Fe levels seem to be unaffected in this mutant compared to wildtype worms (Supplementary). In addition to neurodegeneration, obesity and steatosis have been reported in ceruloplasmin-KO mice (Raia et al., 2023), highlighting that ceruloplasmin is essential for Cu and Fe homeostasis. Our data revealed that Cu induced *ceruloplasmin* mRNA expression in wildtype and atox-1 deletion mutants (Figure 7), possibly due to the excretion of excess Cu bound to ceruloplasmin. In addition, excess Cu is excreted by atp7b, which in humans, among others, participates in providing Cu to ceruloplasmin (Prohaska, 2008).

In mammals, the two major functions of ATP7B are the supply of Cu to ceruloplasmin in the golgi and the excretion of excess Cu into the bile (Weiss et al., 2008). ATP7B translocalizes to the plasma membrane which enables the efflux of excess Cu in the form of vesicular sequestration (Monty et al., 2005; Cater et al., 2006). This aligns with our data where *atp7a/b* mRNA levels were elevated in wildtype worms following Cu treatment. Similar observations were made by Li et al. after Cu treatment (Li et al., 2021). The notion that ATP7B is essential for proper functioning Cu homeostasis is supported by experiments in ATP7B-KO models (Muchenditsi et al., 2021). Our data reveal an increase of *atp7a/b* mRNA levels due to Cu treatment in wildtype worms, but further display that untreated atox-1 and ceruloplasmin deletion mutants already exhibit elevated *atp7a/b* levels (Figure 7). Interestingly, Cu treatment does not increase *atp7a/b* in those mutants further. The fact that atox-1Δ and ceruloplasminΔ mutants demonstrate greater *atp7b* expression but lower total Cu levels compared to wildtype worms is unexpected. This suggests that one should not focus exclusively on total Cu levels, but also on labile Cu levels, which differ among the worms used in this study and are notably increased in atox-1 and ceruloplasmin deletion mutants.

Traditionally, Cu status is assessed by measuring serum or plasma total Cu and ceruloplasmin levels (Olivares et al., 2008; Hackler et al., 2020), whereas for WD diagnosis a liver biopsy is required (Mohr and Weiss, 2019). Besides ceruloplasmin protein levels, its enzyme activity and mRNA level also affect the maintenance of Cu homeostasis (Ranganathan et al., 2011). Furthermore, labile Cu has recently emerged as a marker of Cu status, as it is assumed to be readily bioavailable and reflects more accurately Cu activity compared to total Cu (Dodani et al., 2014; Kardos et al., 2018). Our data reveal that atox-1Δ and ceruloplasminΔ mutants displayed reduced total Cu levels compared to wildtype worms following Cu treatment, as well as severe alterations of Cu homeostasis, e.g., increased mRNA levels of *atp7a/b*. This could be linked to elevated levels of labile Cu. Nevertheless, relying on labile Cu levels is currently not considered sufficient due to the complexity of analysis and lack of methodologies available (Cotruvo et al., 2015; Xiao et al., 2018; Quarles et al., 2020; Pezacki et al., 2022). Having said that, in combination with total Cu and ceruloplasmin measurements, the analysis of labile Cu promises to be a valuable and powerful tool to assess the Cu status and thus the risk or diagnosis for Cu dyshomeostasis-related diseases (Shribman et al., 2021). Squitti et al. observed a subpopulation of patients diagnosed with Alzheimer’s disease (AD) displaying higher than normal non-ceruloplasmin bound Cu in serum, similar to WD patients, stating that labile Cu identifies a Cu subtype of AD (CuAD) (Squitti et al., 2021; Squitti et al., 2023). They further hypothesize that Cu dyshomeostasis results in a shift of protein-bound metal pools to labile metal pools, which is associated with the loss of energy production but also altered antioxidant function (Squitti et al., 2021). Labile Cu pools are increased even by physiological Cu amounts in different brain cells (Lee et al., 2020), which can exert neurotoxicity (Ugarte et al., 2013). Elevated Cu levels promote the formation of reactive oxygen species, lipid peroxidation, apoptosis and decreased mitochondrial membrane potential, leading to oxidative stress (Song et al., 2014; Li et al., 2021). Borchard et al. report that labile Cu is cell toxic with mitochondria as vulnerable target, which in turn can be avoided by Cu chelation (Borchard et al., 2022). Several studies reveal that Cu chelation reduces or even prevents Cu-mediated toxicity (Lichtmannegger et al., 2016; Yuan et al., 2018; Yuan et al., 2022), while studies in WD models demonstrate that chelation lowers systemic Cu levels into the homeostatic range as possible therapeutic approach (Brady et al., 2014; Müller et al., 2018; Einer et al., 2023). Chelator therapy also suggests that Cu toxicity is mainly mediated by labile Cu rather than by pre-protein bound Cu like in ceruloplasmin. Local concentrations of Cu as well as cellular distribution of Cu transporters, storage and excretion proteins are important to maintain a steady state (An et al., 2022) and tight regulation of Cu homeostasis, as dyshomeostasis is associated with the pathogenesis of neurodegenerative diseases such as WD, but also AD (Mezzaroba et al., 2019; Bisaglia and Bubacco, 2020; Chen et al., 2022).

Metallothionein binds excess metal ions to maintain homeostasis, thus the downregulation of *mtl-1* mRNA levels after Cu treatment (Figure 7) seems surprising but supports Zhang et al. who too report a reduction of metallothionein mRNA levels in Cu exposed *C. elegans* (Zhang et al., 2021). In contrast, Tapia et al. were not able to identify alterations in metallothionein mRNA levels in rat fibroblast cells (Tapia et al., 2004), which suggests either the presence of tissue- or species-specific differences in metallothionein transcription. *C. elegans* metallothioneins seem to be strongly induced by other trace elements, such as zinc (Polak et al., 2014; Baesler et al., 2021) as well as cadmium (Hughes et al., 2009; Zeitoun-Ghandour et al., 2010; Essig et al., 2023). Notably, mRNA levels are not necessarily proportional to protein levels (Buccitelli and Selbach, 2020), this also applies to metallothionein (Michaelis et al., 2022). Our data suggest slight changes in mtl-1, but not mtl-2 expression. Since mtl-2 is present in larger quantities in *C. elegans* (Zeitoun-Ghandour et al., 2010; Höckner et al., 2011), alterations in mtl-1 are marginal with respect to total metallothionein levels. Nevertheless, mtl-1 still seems to be important for metal uptake, as mtl-1 knockouts take up less Cu and other trace elements in our but also previous studies (Tapia et al., 2004; Baesler et al., 2021). Additionally, metallothionein preserves Cu-induced neurotoxicity (Petro et al., 2016).

In summary, we were able to uncover that ceruloplasmin and atox-1 play a key role in Cu homeostasis in *C. elegans*. ICP-OES and ToF-SIMS analysis revealed that total Cu levels were reduced in the ceruloplasmin and atox-1 deletion mutants compared to wildtype worms, in contrast they displayed increased levels of labile Cu. Furthermore, ToF-SIMS analysis is a powerful tool applied in this study enabling firstly a 3D Cu localization in worms. Accordingly, a genetic Cu dyshomeostasis and Cu oversupply can result in a shifted ratio of total Cu vs. labile Cu pools. The dyshomeostasis is further reflected by an altered gene expression of crucial participants in Cu homeostasis like *atp7a/b*, *atox-1*, *ceruloplasmin* and *metallothionein*. As demonstrated here, labile Cu is a potential marker of the Cu status in the organism *C. elegans*. Taken together, the *C. elegans* genome encodes a suite of evolutionary conserved genes involved in Cu homeostasis and thus serves as an exquisite model to study Cu dyshomeostasis linked to neurodegenerative diseases. However, some aspects remain unanswered and require further investigation, such as the mechanistic regulation of atox-1 and ceruloplasmin in *C. elegans*. Our study demonstrates early observations of a defective Cu homeostasis in *C. elegans*, but also reveals a lack of knowledge of underlying mechanisms due to complexity, which should be addressed in future studies. Altogether, the Cu status should be monitored by multiple functional markers including total Cu, labile Cu as well as gene expression of Cu homeostasis-related genes in order to provide specificity and sensitivity to detect potential alterations in Cu homeostasis.

## Data Availability

The original contributions presented in the study are included in the article/Supplementary Material, further inquiries can be directed to the corresponding author.
