# Supplementary material for: Dysfunction in atox-1 and ceruloplasmin alters labile Cu levels and consequently Cu homeostasis in C. elegans
Source: Front Mol Biosci. 2024 Feb 8;11:1354627. doi: 10.3389/fmolb.2024.1354627 (PMC10882093; doi:10.3389/fmolb.2024.1354627)
Supplement: Supplementary file 1 [file DataSheet1.PDF]

Supplementary Material

1 Supplementary Figures and Tables

1.1 Supplementary Figures

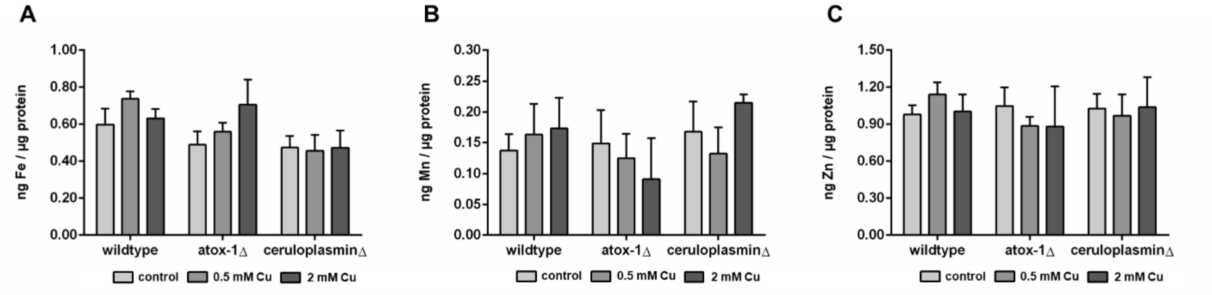

Figure S1: Total (A) Fe, (B) Mn and (C) Zn levels quantified by ICP-OES in wildtype, atox-1Δ and ceruloplasminΔ mutants following 24 h Cu treatment. Data presented are mean values of $n \geq 4$ independent experiments + SEM.

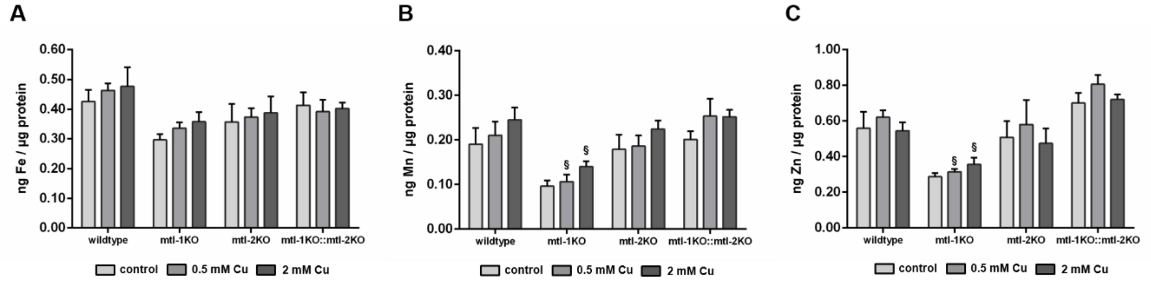

Figure S2: Total (A) Fe, (B) Mn and (C) Zn levels quantified by ICP-OES in wildtype, mtl-1KO, mtl-2KO and mtl-1KO::mtl-2KO mutants following 24 h Cu treatment. Data presented are mean values of $n = 4$ independent experiments + SEM. Statistical analysis using 2-way ANOVA with Tukey's multiple comparison. Significance level with $\alpha = 0.05$: *: $p \leq 0.05$; compared to untreated control and §: $p \leq 0.05$; §§: $p \leq 0.01$; §§§: $p \leq 0.001$ compared to wildtype in same condition.
